# Investigating the Vascularization of Tissue-Engineered Bone Constructs Using Dental Pulp Cells and 45S5 Bioglass^®^ Scaffolds

**DOI:** 10.1089/ten.tea.2014.0485

**Published:** 2015-04-27

**Authors:** Reem El-Gendy, Jennifer Kirkham, Phillipa J. Newby, Yamuna Mohanram, Aldo Roberto Boccaccini, Xuebin B. Yang

**Affiliations:** ^1^ Biomaterials and Tissue Engineering Group, Department of Oral Biology, University of Leeds, St. James's University Hospital, Leeds, United Kingdom.; ^2^ Biomineralisation Group, Department of Oral Biology, University of Leeds, St. James's University Hospital, Leeds, United Kingdom.; ^3^ Department of Oral Pathology, Faculty of Dentistry, Suez Canal University, Ismailia, Egypt.; ^4^ Department of Materials, Imperial College London, London, United Kingdom.; ^5^ Institute of Biomaterials, University of Erlangen-Nuremberg, Erlangen, Germany.

## Abstract

Identification of a suitable cell source combined with an appropriate 3D scaffold is an essential prerequisite for successful engineering of skeletal tissues. Both osteogenesis and angiogenesis are key processes for bone regeneration. This study investigated the vascularization potential of a novel combination of human dental pulp stromal cells (HDPSCs) with 45S5 Bioglass^®^ scaffolds for tissue-engineered mineral constructs *in vivo* and *in vitro*. 45S5 Bioglass scaffolds were produced by the foam replication technique with the standard composition of 45 wt% SiO_2_, 24.5 wt% Na_2_O, 24.5 wt% CaO, and 6 wt% P_2_O_5_. HDPSCs were cultured in monolayers and on porous 45S5 Bioglass scaffolds under angiogenic and osteogenic conditions for 2–4 weeks. HDPSCs expressed endothelial gene markers (*CD34, CD31/PECAM1, and VEGFR2*) under both conditions in the monolayer. A combination of HDPSCs with 45S5 Bioglass enhanced the expression of these gene markers. Positive immunostaining for CD31/PECAM1 and VEGFR2 and negative staining for CD34 supported the gene expression data, while histology revealed evidence of endothelial cell-like morphology within the constructs. More organized tubular structures, resembling microvessels, were seen in the constructs after 8 weeks of implantation *in vivo*. In conclusion, this study suggests that the combination of HDPSCs with 45S5 Bioglass scaffolds offers a promising strategy for regenerating vascularized bone grafts.

## Introduction

Vascularization of tissue-engineered constructs is essential to allow diffusion of oxygen and nutrients to the constructs' center, avoiding lack of perfusion that might otherwise lead to central necrosis.^1^ It is well known that vascularization plays a major role in endochondral and intramembranous ossification and is a central requirement for bone regeneration and fracture healing. Angiogenesis during bone regeneration is mediated through the angiopoietin or the vascular endothelial growth factor (VEGF) pathway, with the latter known to be the most important.^2^ The identification of appropriate cells and scaffolds that will allow/promote angiogenesis and vascular growth within the constructs is therefore the determining factor for future clinical applications in bone tissue engineering.^3^

Human dental pulp stem/stromal cells (HDPSCs) are a population of cells present in the pulp, the vital part of adult and deciduous teeth. They are multipotent, highly proliferative, and capable of producing mineralized nodules *in vitro* or forming a pseudo pulp/dentin complex and/or bone *in vivo* depending on the site of implantation.^4–6^ HDPSCs are known to express several stem cell surface antigen markers, among which are vascular-associated and smooth muscle markers, such as vascular cell adhesion molecule (VCAM), alpha smooth muscle actin, and melanoma-associated antigen/mucin-18 (MUC-18)/CD146, which identifies stem cells of the endothelial lineage. The expression of such markers was thought to be associated with the perivascular niche origins of HDPSCs.^7,8^ HDPSCs regenerate and repair the dental pulp tissue complex. Thus, they are potentially predisposed to default toward angiogenic differentiation.^8–11^

In light of the importance of angiogenesis in wound healing^12^ and in both endochondral and intramembranous ossification,^13^ the angiogenic potential of HDPSCs in relation to their osteogenic differentiation has been further investigated. Many researchers have been interested in HDPSC-associated angiogenesis as an essential pillar for pulp regeneration,^5,14^ others have investigated the use of the angiogenic potential of these cells in treating ischemic limb disease.^15^ However, fewer studies have looked at the combination of their osteogenic/angiogenic properties.^16^

Silicate bioactive glasses, first investigated by Hench *et al.*,^17^ have been well researched as 3D bone tissue scaffolds. The application of bioactive glasses and glass-ceramics in bone tissue engineering is expanding.^18,19^ Furthermore, bioactive glasses can also serve as carriers for the local delivery of metal ions to control cellular functions.^20^ The dissolution products from such glasses can upregulate expression of genes that control osteogenesis.^21,22^ In addition, there is increasing evidence of the positive effects of bioactive glass on vascularization of tissue engineering constructs.^22,23^ The angiogenic potential of 45S5 Bioglass^®^ has been reported previously, but is not yet well established.^24^

In the present study, we have investigated the expression of endothelial cell markers by HDPSCs in monolayer culture, 3D culture (in combination with porous 45S5 Bioglass scaffolds), and after *in vivo* implantation to test the hypothesis that this novel combination might promote and support construct vascularization.

## Materials and Methods

Cell culture plastics were purchased from Corning. Alpha-modified minimum essential medium (α-MEM), phosphate-buffered saline solution, and fetal bovine serum (FBS) were obtained from Lonza. Antibiotics, growth factors, enzymes, and other reagents were purchased from Sigma, unless stated otherwise.

### Structural characterization of 45S5 Bioglass scaffolds

The starting bioactive glass powder used in these investigations (45S5 Bioglass) had the standard composition of 45 wt% SiO_2_, 24.5 wt% Na_2_O, 24.5 wt% CaO, and 6 wt% P_2_O_5_.^17^ Scaffolds were produced by the foam replication technique as described by Chen *et al.*^25^ and El-Gendy *et al.*^26^ The sintered scaffolds produced for this work were subjected to a range of characterization techniques to ensure that they were comparable with previous work and were suitable for use in bone tissue engineering.

The scaffolds were examined in the scanning electron microscope (SEM) to determine the pore dimensions, strut cross section, and the topography of the scaffold using a JEOL JSM 5610LV SEM. The overall porosity of the scaffold was determined using a measure of the scaffold's physical dimensions, its mass, and the density of the Bioglass powder. The crystalline structure of the scaffold was measured through X-ray diffraction (XRD) using a Phillips PW1700 series machine using Cu Kα radiation and the resulting data were processed using X'Pert HighScore combined with the PCPDF data base.

The chemical structure of the scaffold was assessed by deploying Fourier transform infrared spectroscopy (FTIR) using a Bruker Vector 22 TGA-IR setup to measure transmission spectra. The mechanical competence of the scaffolds was measured by ascertaining the compressional strength using a Zwick testing machine. Surface topography was investigated using white light interferometry (WLI) and wettability using a Zygo NewView™ 200 white light microscope-based interferometer and a Kruss DSA30 instrument respectively. The measurements were carried out on cylindrical pellets fabricated using the same conditions employed to fabricate the scaffolds. The bioactivity of the scaffolds was also assessed by soaking the scaffolds in simulated body fluid (SBF) for 14 days, prepared in accordance with Kokubo,^27^ and characterized using the techniques described previously in this section to determine if hydroxyapatite was present as this is the marker of the bioactive character of the scaffold.

### Cell isolation and *in vitro* expansion

The pulp tissues were collected from three wisdom teeth from three different donors (1 male, 19 years old and 2 females, 20 and 37 years old), with full patient consent and ethical approval (LREC 07/H1306/93). HDPSCs were isolated using the collagenase digestion method as previously described.^4,28^ The cells were maintained in basal medium (α-MEM supplemented with 20% FBS, 200 mM l-glutamine, and 100 units/mL penicillin/streptomycin) at 37°C and 5% CO_2_ until 80% confluent.^26^ Passage 4 (P4) cells were used for this study.

### Human dental pulp stromal cell culture as monolayers *in vitro*

HDPSCs were seeded into six-well plates at 5×10^5^ cells per well (*n*=3 for each of three donors) and cultured under basal or osteogenic (basal medium supplemented with 100 nM dexamethasone and 50 μM of l-ascorbic acid-2 phosphate) conditions for up to 2 and 4 weeks. The samples were then collected for quantitative real-time PCR (qRT-PCR) to look at endothelial gene expression.

### Human dental pulp stromal cell seeding and growth on 3D scaffolds

HDPSCs (5×10^5^ cells/scaffold) were dynamically seeded and cultured on sterile 3D Bioglass scaffolds (5×5×5 mm^3^) for 5 days using an in-house rotating bioreactor, as previously described.^26^ The cell–scaffold constructs were then statically cultured in basal or osteogenic conditions (*n*=3/donor) at 37°C and 5% CO_2_ for 2 and 4 weeks with weekly changes of the medium.

### Comparing endothelial gene expression of HDPSCs in monolayers and in 3D cultures using qRT-PCR

Expression of endothelial marker genes (*CD34, CD31/PECAM1,* and *VEGFR2*) was assessed using qRT-PCR. *GAPDH* was used as the housekeeping gene. RNA was extracted using the TRIzol reagent kit (Invitrogen) according to the manufacturer's instructions. One microgram of RNA from each sample was used for reverse transcription using the ABI High-Capacity RNA-to-cDNA kit (Applied Bioscience) according to the supplier's instructions. cDNA was then amplified using ABI TaqMan primers (*GAPDH*: Hs99999905-m1, *CD34*: Hs00990732-m1, *CD31/PECAM1*: Hs01065279-m1, *VEGFR2*: Hs00911700-m1) in a 20 μL reaction mix in 96-well plates (Roche). Amplification was performed using a Roche LC480 cycler.

The results were analyzed using the 2^−ΔΔct^ method^29^ where ct values at each time point were normalized to the housekeeping gene in the same sample and further normalized to ct values of control samples (cultured under basal conditions) at the corresponding time points. Results were then expressed as log mean 2^−ΔΔct^±standard deviation (SD).

### *In vivo* intraperitoneal implantation of 3D constructs (45S5 Bioglass scaffolds seeded with HDPSCs)

The diffusion chamber model provides an enclosed space within the host animal for studies of cellular proliferation/differentiation of implanted human cells. The filters on the diffusion chamber allow free exchange of nutrients (including many well-known and/or unknown growth factors, which may be crucial for functional tissue engineering) and waste, but effectively isolate the experimental cells from the host tissues.^30,31^ The *in vivo* experiments were carried out in strict accordance with ethical guidelines and UK Home Office regulations under a project license.

The 3D45S5 Bioglass scaffolds seeded with HDPSCs were used as the test group (*n*=4), and scaffolds without cells were used as the negative controls (*n*=2). The constructs (together with the control scaffolds) were dynamically cultured for 5 days, followed by static culture in basal medium for a further 2 days, before being sealed into diffusion chambers (Millipore) and implanted intraperitoneally in male immunocompromised nude mice (MF1-Nu/Nu, 4–5 weeks old).^32^ Mice were sacrificed after 8 weeks according to UK Home Office regulations. The diffusion chambers were retrieved and samples were fixed in 10% neutral buffered formalin before processing for immunohistochemistry.

### Immunohistochemical examination of cell–scaffold constructs

Samples from both *in vitro* and *in vivo* studies were paraffin embedded and sectioned at 5 μm. Sections were stained for immunohistochemistry using primary antibodies directed against human cluster of differentiation 34 (CD34, mouse monoclonal, ab8536, 1/100; Abcam), platelet endothelial cell adhesion molecule 1 (PECAM1, also known as CD31, rabbit polyclonal, ab28364, 1/100; Abcam), and vascular endothelial growth factor 2 (VEGFR2, rabbit polyclonal, ab28364, 1/100; Abcam). The Envision kit (Dako) was used to provide secondary antibodies and substrate in each case. Negative controls, which were not exposed to primary antibodies, were included in the study.

Three random fields from each section and three sections from each sample were recorded and assessed by a blinded operator. The stain intensity was then semiquantitatively assessed. Microvessel-like tubular structures were categorized according to the diameters of their lumens (<40 and >40 μm), which was due to some capillaries (such as sinusoidal capillaries) having larger openings (30–40 μm in diameter), although most of the capillaries are about 5–10 μm in diameter.^33–35^ Immunopositive microvessel-like tubular structures were counted and recorded for each marker^36^ using NIS-Elements BR 3.0 software.

### Statistical analyses

Each experiment was repeated thrice using cells from three different donors. Results shown are presented from one representative donor.

qRT-PCR data were statistically analyzed using a one-way ANOVA test, followed by Bonferroni multiple comparison tests. The statistical analyses were carried out using the GraphPad Instat software (version 3).

Microvessel-like tubular structure counts were tested for statistical significance using two-way ANOVA testing (GraphPad Prism: version 6).

## Results

### Characterization of the 45S5 Bioglass scaffolds in preparation for the cell culture work

A standard set of characterization techniques was used to investigate the properties of these 45S5 Bioglass scaffolds to ascertain whether they were suitable for the cell culture work and to prove that they aligned with previous results.^25,26^

**FIG. 1.**
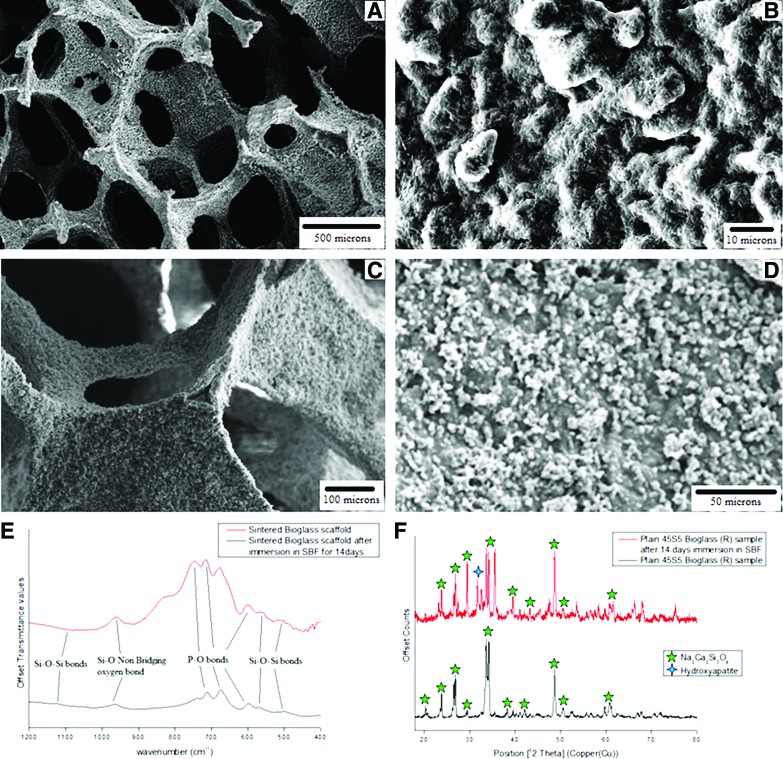
Structural characterization of 45S5 Bioglass^®^ scaffolds using a scanning electron microscope and spectra analysis. **(A)** The overall porosity of the scaffold, **(B)** the microtopography of the surface of a scaffold, **(C)** the cross section of a strut from a 45S5 Bioglass scaffold, **(D)** the presence of hydroxyapatite on the surface of the scaffold after immersion in simulated body fluid (SBF) for 14 days, **(E)** Fourier transform infrared spectroscopy spectra before and after immersion in SBF for 14 days, and **(F)** X-ray diffraction spectra before and after immersion in SBF for 14 days. Color images available online at www.liebertpub.com/tea

The porosity of the sintered scaffold was found to average above 90% with a pore size ranging between ∼200 and 600 μm with a cross-sectional strut size in the range of 25–75 μm, as seen in Figure 1A and C, respectively, consistent with previously published work on silicate scaffolds fabricated by the same foam replica technique.^25,37^

Figure 1B shows that the surface of the scaffold had a rough microtopography, which in previous studies has been shown to encourage cell attachment^38^ and, given the increased surface area, accelerates surface reactions.^39^ The roughness seen in Figure 1B is further supported by the WLI and wettability studies. The WLI study produced average root-mean-square roughness (RMS), roughness average (Ra), and peak-to-valley (PV) surface roughness values of 3.3±1.3, 2.6±0.9, and 19.1±9.0 μm, respectively, and the wettability study produced a contact angle of 28°±4°. Both sets of results are in agreement with results in the literature.^40^

Figure 1D shows the effects of immersion in SBF for 14 days on the surface of the scaffold with the presence of hydroxyapatite deposits and a decrease in the scaffold's porosity to ∼83%. This effect changes the surface topography with the WLI study showing RMS, Ra, and PV values of 6.3±1.4, 5.7±1.1, and 25.5±8.7 μm, respectively, and the contact angle from the wettability study being 23°±3° after immersion in SBF for 14 days; both values fall in line with the previous experimental results.^41^

The mechanical competence of the 45S5 Bioglass scaffold was assessed using compressive strength tests, which gave an average mechanical strength of 0.53±0.08 and 0.43±0.05 MPa before and after immersion in SBF, respectively, which is again consistent with data presented in previous studies.^25,42,43^

Figure 1E shows the FTIR spectra of sintered 45S5 Bioglass scaffolds before and after immersion in SBF, and characteristic bond peaks^44,45^ are clearly seen in both spectra. However, the scaffold that was immersed in SBF for 14 days exhibited signs of hydroxyapatite formation as seen by the changing shape of the spectra, which is in agreement with previously published results.^46^

Figure 1F shows the XRD spectra before and after immersion in SBF for 14 days and confirms the crystalline nature of the scaffolds with the main crystalline phase being Na_2_Ca_2_Si_3_O_9_, which is consistent with previous investigations.^25,47,48^ The spectrum corresponding to the scaffold after immersion in SBF for 14 days shows the presence of hydroxyapatite, indicating that the samples are bioactive and therefore suitable for bone tissue engineering applications.^41^

Overall, the fabricated foam replicated 45S5 Bioglass-derived glass–ceramic scaffolds are consistent with the state of the art for this type of biomaterial and they were therefore selected to be used in the cell culture section of this study.

### The effect of 3D 45S5 Bioglass scaffolds on the expression of vascular/endothelial marker genes in HDPSC-Bioglass constructs *in vitro*

**FIG. 2.**
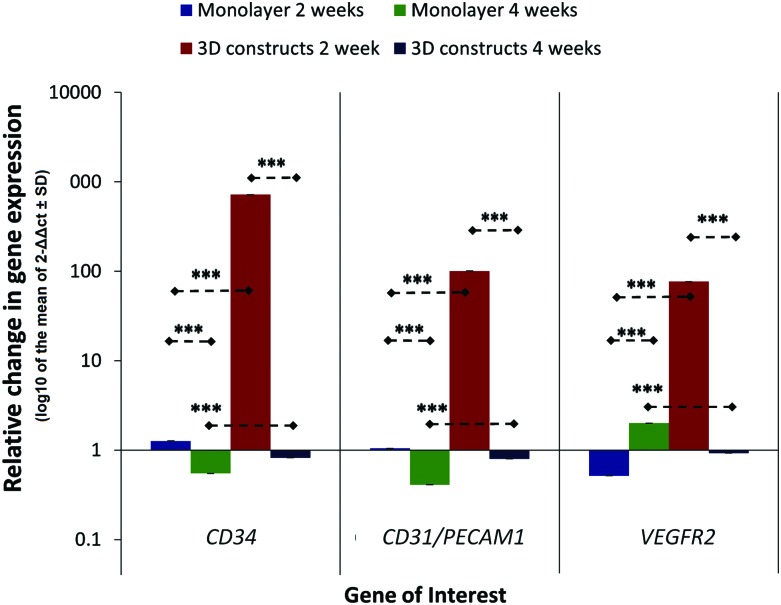
Relative change in the levels of angiogenic marker gene expression between human dental pulp stromal cells (HDPSCs) cultured in monolayers and in 3D 45S5 Bioglass for 2 and 4 weeks *in vitro*. The data are from HDPSCs under osteogenic conditions, which were normalized to corresponding control samples cultured under basal conditions. These are presented as log10 of the mean of 2^−ΔΔct^±standard deviation. ***p<0.001. Color images available online at www.liebertpub.com/tea

To determine the effect of the 3D 45S5 Bioglass scaffolds on endothelial marker gene expression by HDPSCs, the levels of gene expression for HDPSCs cultured under osteogenic conditions as monolayers were compared with those for HDPSCs on 3D 45S5 Bioglass scaffolds. The expression was normalized to corresponding control samples cultured under basal conditions at 2 and 4 weeks (Fig. 2). The results are presented in the form of mean log_10_ 2^−ΔΔct^±SD as explained earlier. The HDPSC expression levels of all three endothelial markers (*CD34, CD31/PECAM1, and VEGFR2*) were significantly higher for cells in 3D 45S5 Bioglass constructs compared with the expression of the same markers by HDPSCs cultured in monolayers (*p*<0.001). There was a significant decrease in *CD34* and *CD31/PECAM1* gene expression for HDPSCs cultured in both 3D constructs and as monolayers at 4 weeks compared with the expression levels of these genes at 2 weeks (*p*<0.001). In spite of this decrease, the levels of expression for *CD34* and *CD31/PECAM1* were still significantly higher (*p*<0.001) in 3D constructs compared with monolayer cultures at the same time point. However, *VEGFR2* showed significant upregulation (*p*<0.001) in HDPSCs cultured in monolayers for 4 weeks compared with the downregulation observed in cells cultured in the 3D constructs.

### Distribution of positive specific endothelial markers within HDPC-3D 45S5 Bioglass scaffold constructs cultured *in vitro* and *in vivo*

**FIG. 3.**
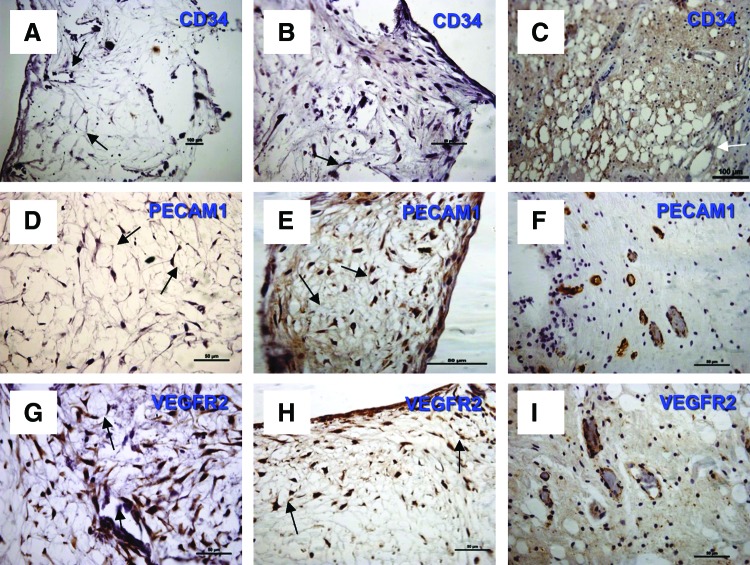
Immunohistochemical localization of angiogenic markers (CD34, PECAM1, and VEGFR2) in cell–scaffold constructs cultured *in vitro* for 6 weeks. Light microscope images showing immunohistochemical staining for cell–scaffold constructs cultured under basal conditions **(A, D, G)** or under osteogenic conditions **(B, E, H)** *in vitro*. Human pulp tissues were used as the positive control **(C, F, I)**. **(A–C)** CD34 antibodies, **(D–F)** PECAM1 antibodies, and **(G–I)** VEGFR2 antibodies. *Arrows* pointing to flat endothelial-like cells lining capillary-like structures in HDPS-scaffold constructs. All sections were counterstained with Harris' hematoxylin. Scale bar=50 μm, except **(A)**, for which the scale bar=100 μm. Color images available online at www.liebertpub.com/tea

The distribution of positive immunostaining for endothelial markers within the cell–scaffold constructs following *in vitro* culture under basal and osteogenic conditions is shown in Figure 3. Positive staining was detected for all three markers in both the basal (Fig. 3A, D, G) and osteogenic (Fig. 3B, E, H) culture groups, as well as for the positive controls (Fig. 3C, F, I). Positively stained cells demonstrated a flat fibroblastic phenotype rather than the cuboidal or polygonal cell morphology that might be expected to indicate a more vascular phenotype. They also aligned to form microvessel-like tubular structures (capillary-like structures), which seemed to be less developed than the obvious capillary-like structures seen in the positive control (pulp tissue) (Fig. 3C, F, I).

Stronger positive immunostaining was detected in HDPSC-scaffold constructs cultured under osteogenic conditions, while weaker positive staining was detected under basal conditions for all endothelial markers (Fig. 3 and Table 1).

**Table 1.** Immunohistochemical Stain Intensity Based Upon Blind Assessment

	In vitro		
*Marker*	*Basal*	*Osteogenic*	In vivo
CD34	−	+	−
PECAM1/CD31	−/+	++	+/++
VEGFR2	++	++/+++	+

**FIG. 4.**
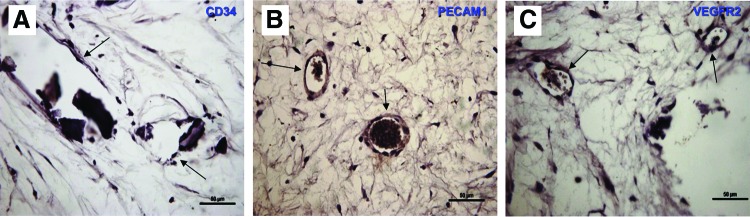
Immunohistochemical localization of angiogenic markers in a cell scaffold implanted intraperitoneally in nude mice *in vivo* for 8 weeks. Light microscope images showing immunohistochemical staining of cell–scaffold constructs implanted intraperitoneally in nude mice **(A–C)**. **(A)** CD34, **(B)** PECAM1, **(C)** VEGFR2. *Arrows* pointing to flat endothelial-like cells lining capillary-like structures. All sections were counterstained with Harris' hematoxylin. Scale bar=50 μm. Color images available online at www.liebertpub.com/tea

The presence and distribution of the endothelial proteins, CD34, CD31/PECAM1, and VEGFR2, in HDPSC-scaffold constructs implanted intraperitoneally in immunocompromised nude mice for 8 weeks was also determined using immunohistochemistry. CD34 showed the weakest staining intensity compared with CD31/PECAM1 and VEGFR2 markers in all of the constructs (Table 1). Many of the positively stained cells seemed to align with more well-developed microvessel-like tubular structures (Fig. 4) that were comparable with those seen in the positive control (pulp tissue) (Fig. 3C, F, I).

**FIG. 5.**
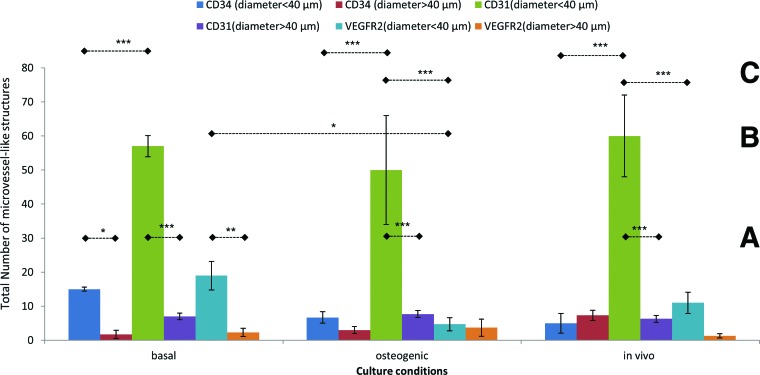
The total number of small (<40 μm diameter) and large (>40 μm diameter) microvessel-like tubular structures that were positively stained for CD34, CD31, and VEGFR2 in HDPSC-Bioglass constructs under *in vitro* (basal/osteogenic) and *in vivo* conditions. **(A)** Comparison of the positive staining for same angiogenic markers between small (<40 μm) and large (>40 μm)-diameter microvessel-like structures under the same culture conditions; **(B)** comparison of the positive staining for same angiogenic markers of similar diameter microvessel-like structures under different culture conditions; **(C)** comparison of the positive staining of the three different angiogenic markers of similar diameter microvessel-like structures under same culture conditions. *p<0.05, **p<0.01, ****p<0.001. Color images available online at www.liebertpub.com/tea

The microvessel-like tubular structures were categorized according to their lumen size and enumerated for each of the markers for all culture conditions *in vitro* and *in vivo* (Fig. 5A). When comparing the same positively stained different sizes of microvessel-like tubular structures within the same culture condition (Fig. 5A), the number of smaller (S) microvessel-like tubular structures (<40 μm diameter) stained positively for CD 31 was significantly higher than the positively stained larger (L) microvessel-like tubular structures (>40 μm diameter) (*p*<0.001) in both basal and osteogenic culture conditions and from the *in vivo* study. Similarly, there were significantly higher positive CD34 (*p*<0.05) and VGFR2 (*p*<0.01)-stained smaller microvessel-like tubular structures than the larger one in the basal medium culture conditions. However, there were no significant differences in the osteogenic culture and *in vivo* condition (*p*>0.05).

When comparing the same positively stained and same size of microvessel-like tubular structures between different culture conditions (Fig. 5B), there was significantly higher number of VEGFR2-positive-stained smaller microvessel-like tubular structures in the basal culture condition than that in the osteogenic culture condition (*p*<0.05). There were no significant differences between other comparisons (Fig. 5B).

When comparing the same size of microvessel-like tubular structures in the same culture condition between different stainings (Fig. 5C), there was significantly less number of CD34-positive-stained smaller microvessel-like tubular structures than the CD31-positive-stained ones in basal, osteogenic, and *in vivo* conditions (*p*<0.001). Similarly, there was significantly less number of VEGFR2-positive-stained smaller microvessel-like tubular structures than the CD31-positive-stained ones in only osteogenic and *in vivo* conditions (*p*<0.001). There were no significant differences between other comparisons (Fig. 5C).

## Discussion

In the present study, foam replicated sintered 45S5 Bioglass scaffolds were produced and characterized to assess their suitability for use in cell culture studies and ultimately in bone tissue engineering. The characterization tests showed that the foam replication technique produced scaffolds of suitable structural quality with convenient porosity to allow cells to pass through the scaffold while maintaining structural integrity to avoid collapse after a short immersion period in SBF. These results uphold previous work that showed that scaffolds fabricated by the foam replication technique are able to be easily produced in large quantities in a variety of shapes and sizes,^25^ bioactive,^25^ partially crystalline,^48^ mechanically competent,^25,42^ have a suitable porosity,^25^ a surface topography that promotes cell attachment,^40,42–44^ and a chemical structure, which lends itself to the formation of hydroxyapatite once immersed in SBF.^44,45^ There are increasing research efforts dedicated to understanding the effect that such bioactive glass scaffolds have on vascularization of the constructs for bone tissue engineering applications.^26^

Previously, we have demonstrated the effect of 45S5 Bioglass on osteogenic differentiation of HDPSCs.^26^ In this study, we first determined gene expression of endothelial markers by HDPSCs in monolayer culture. The resulting pattern of endothelial marker gene expression may be attributable to a number of factors. The early upregulation of *CD34 (*considered by some to be hematopoietic and an early endothelial cell marker, as well as being an early transdifferentiation marker of angiogenesis) is an indication of early angiogenic differentiation.^49^ This correlates well with the lower levels of expression seen for *CD31/PECAM1* and *VEGFR2* at 2 weeks and the upregulation of *VEGFR2* at 4 weeks. The downregulation of these markers under osteoinductive conditions at 2 weeks may also be attributed to the reported angiogenic inhibitory effects of dexamethasone and AA2P, which are both present in the osteoinductive culture medium.^17,18,26,50^

It might also be that the pattern of gene expression observed in monolayer culture is related to the lack of any scaffold to provide an appropriate 3D microenvironment. For this reason, endothelial marker gene expression was investigated for HDPSCs cultured in 45S5-based Bioglass constructs under osteogenic conditions and this was compared with expression in similar controls cultured under basal conditions. All endothelial markers (*CD34, CD31/PECAM1*, and *VEGFR2*) were upregulated at 2 weeks and downregulated at 4 weeks compared with the basal culture controls, suggesting a more advanced angiogenic differentiation stage for HDPSCs on 3D 45S5 Bioglass scaffolds. Although *CD34* is generally thought to be indicative of early differentiation,^49^ *CD34* expression was reported to be variable, with different expression levels being reported in different tissues; and even with the different size of a given blood vessel,^51^ *CD31/PECAM1* and *VEGFR2* are both considered to be markers for endothelial differentiation, proliferation, and expansion.^52^ Thus, their upregulation at 2 weeks in the present study suggests a more advanced stage of angiogenic differentiation compared with cells cultured under the same culture conditions in the monolayer. This was followed by a decrease in expression levels at the later time points, presumably due to a switching off of their expression as the differentiation process advanced.^52^ These data also need to be considered together with the observed significant increase in the expression of osteogenic markers in 3D constructs compared with the monolayer culture at 2 weeks.^26^ This pattern of osteogenic and endothelial marker expression is also seen as a result of signal exchange between endotheliocytes and bone cells. Bone endothelial cells are known to respond to bone regulatory cytokine.^53^

Kanczler and Oreffo suggested that skeletal tissue engineering requires the coculture of endothelial-derived cells as well as osteoprogenitors to ensure adequate vascularization of the construct and integration with the surrounding tissues. They also suggested the use of a bioactive, angiogenic porous scaffold to provide the appropriate cues, where possible, for the use of angiogenic growth factors.^53^ In our case, the combination of a mixed cell population as well as the angiogenic properties of the 45S5 Bioglass scaffold appeared to accommodate these requirements as evidenced by the expression of both osteogenic^26^ and endothelial markers.

The gene expression data were further supported by the histological appearance and immunohistochemical staining of the neotissue produced within the constructs, which showed evidence of endothelial marker expression. For example, under osteogenic culture conditions *in vitro*, enhanced expression of CD31/PECAM1 and VEGFR2 in constructs was seen compared with similar constructs cultured under basal conditions.

HDPSC-scaffold constructs cultured *in vitro* were also seen to contain flattened endothelial-like cells curving into microvessel-like tubular structures redolent of blood vessels. This finding was even more pronounced when HDPSC-scaffold constructs were implanted *in vivo.* Retrieved constructs showed evidence of microvessel-like tubular structures lined with endothelial-like cells that were positively stained for CD31/PECAM1 and VEGFR2 and were weakly positive for CD34 compared with the less developed, microvessel-like tubular structures in their *in vitro* incubated counterparts. This may be attributed to the presence of host cytokines^54^ *in vivo*, although it could also be due to the longer incubation period compared with the *in vitro* constructs. The diffusion chamber is a simple model that allows an enclosed permissive environment to be generated within a host animal while preventing any host tissue participation that might otherwise confound the findings.^55^ This model therefore restricts investigation of test parameters to the implanted cells only. This ensures that any tissue formed in the diffusion chamber is derived exclusively from the test, not the host, cells.^30,31^

The findings presented in this study are in agreement with those of Laino *et al.*, who showed that a CD34-positive population of HDPSCs cultured *in vitro* formed living autologous bone chips,^10^ and d'Aquino *et al.*, who confirmed the angiogenic and osteogenic synergy between osteoblasts and endotheliocytes, both derived from HDPSCs, which produced well-vascularized lamellar bone structures in immunocompromised rats.^11^ We categorized microvessel-like tubular structures/blood vessels in our constructs according to diameter size as less than 40 μm and more than 40 μm. This particular diameter size was selected as it denotes the maximum size for capillaries (fenestrated capillaries).^34^ Our counts showed significantly higher numbers of the smaller-sized vessels compared with larger-sized ones (Fig. 5), indicating good perfusion of the constructs.

The establishment of good vascularity within *in vivo* constructs is of paramount importance for tissue engineering applications as it helps in overcoming the limitation of construct size and necrosis that can occur in large avascularized constructs, one of the major problems that need to be addressed in tissue engineering.^56^

## Conclusion

This study demonstrated the potential of using a combination of a suitable cell source—HDPSCs—with the 45S5Bioglass scaffold to provide a promising functional candidate for vascularized bone tissue-engineered constructs.
